# Prostatic Adenocarcinoma Masquerading as Generalized lymphadenopathy and Mimicking lymphoma on FDG PET/CT: Diagnosis, Staging, and Evaluation of Therapy Response by FDG PET/CT

**DOI:** 10.5812/numonthly.2303

**Published:** 2012-03-01

**Authors:** Prathamesh Joshi, Vikram Lele

**Affiliations:** 1 Department of Nuclear Medicine and PET-CT,Jaslok Hospital and Research Centre, Mumbai, India

**Keywords:** Prostate, Adenocarcinoma, Lymphatic Diseases, Hormone Replacement Therapy, Treatment Response, Positron-Emission Tomography

## Abstract

We report a case of prostatic adenocarcinoma, initially presenting with generalized lymphadenopathy, and mimicking lymphoma on flurodeoxyglucose positron emission tomography/computed tomography (FDG PET/CT). Our case suggests that in elderly men presenting with generalized lymphadenopathy, the diagnosis of metastatic prostatic carcinoma should not be overlooked even in the absence of typical urinary symptoms.The establishment of a diagnosis of metastatic prostate carcinoma is important, because even widespread prostate cancer may be responsive to hormonal treatment, as demonstrated by this case.We also describe the use of FDG PET/CT to diagnose, stage, and evaluate response to hormonal treatment in a given patient.

## 1. Introduction

Metastatic prostate cancer is classically associated with bony or pelvic lymph-nodal metastasis (1). This case report describes an unusual case of prostate carcinoma, presenting with generalized lymphadenopathy, and its appearance on flurodeoxyglucose positron emission tomography/computed tomography (FDG PET/CT). We also describe the valuable role played by FDG PET/CT in diagnosing, staging, and assessing theresponse to therapy in this case.

## 2. Case Report

**Figure 1:**
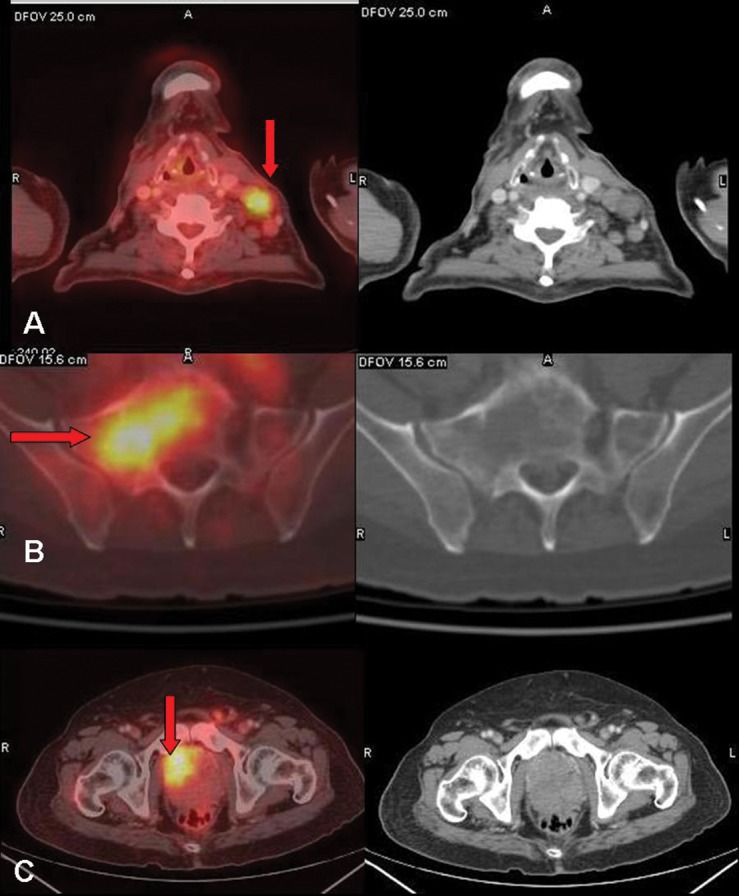
Transaxial Fused FDG PET/CT and CT Images. Figure 1A shows increased FDG uptake in the enlarged left cervical lymph nodes (Arrow), in a lytic lesion in the sacrum (Figure1B arrow), and in the heterogeneously enhancing right lobe of the enlarged prostate gland (Figure1C arrow) with a size of 7.5 × 7.5 cm and maximum standardized uptake value (SUVmax) of 5.5 gm/mL. These findings were indicative of a neoplastic process. TRUS-guided biopsy of the prostate lesion indicated a prostatic adenocarcinoma.

**Figure 2:**
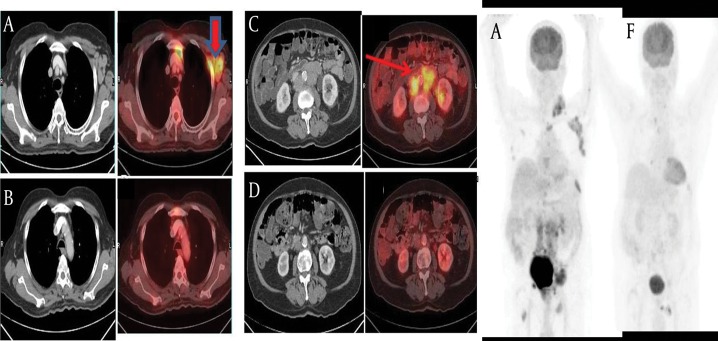
Transaxial CT and Fused FDG PET/CT Images Figure 2A shows multiple enlarged lymph nodes in the left cervical region, with the largest node measuring 2.6 cm in the short axis (SUVmax 6.0 gm/ mL). Figure 2C shows a conglomerate mass of the abdominal lymph nodes with the largest node measuring 6.8 cm in the short axis (SUVmax 5.9 gm/mL). There was complete morphological and metabolic disappearance (Figure 2B and 2D) of these nodes after androgen ablation and hormonal treatment. Maximum intensity projection (MIP) PET images show pretherapy disease burden (Figure 2E) and the dramatic response post-treatment (Figure 2F).

A 70-year-old Indian man presented to his physician with a palpable mass in the left cervical and left axillary region that he had noticed 2 weeks previously, and which had gradually increased in size. On clinical examination and ultrasonography, he was found to have enlarged left cervical, left supraclavicular, and left axillary lymph nodes. He underwent whole-body FDG PET/CT at our center for evaluation of generalized lymphadenopathy. The scan showed increased metabolic activity in multiple enlarged lymph nodes in the left cervical [largest node measuring 2.6 cm in the short axis, maximum standardized uptake value (SUVmax) 6.0 gm/mL;Figure 1A], left supraclavicular (, SUVmax 5.8 gm/mL), left axillary (, SUVmax 6.6 gm/mL;Figure 2A), aortocaval, left para-aortic, retrocaval,preaortic, retro-peritoneal, and bilateral iliac regions( SUVmax of left para-aortic lymph nodes 5.9 gm/mL). The abdominal lymph nodes were forming a conglomerate mass (Figure 2C) compressing and encasing the inferior vena cava, aorta, left renal vein and artery, left upper ureter, and bilateral iliac veins. Lytic-sclerotic lesions with increased metabolic activity were noted in the sternum, right scapula, fifth lumbar vertebra, sacrum (Figure 1B), right iliac bone, left ischial tuberosity, and neck of the right femur, with SUVmax values ranging from 3.0 to 6.0 gm/mL. The scan also revealed metabolically active disease in the heterogeneously enhancing right lobe of the enlarged prostate gland (size 7.5 × 7.5 cm, SUVmax5.5 gm/mL;Figure 1C). In this case, we suspected prostrate carcinoma with skeletal metastases (Stage IV disease) and the possibility of another neoplastic etiology, such as lymphoma, causing FDG accumulation in generalized lymphadenopathy. Serum prostate-specific antigen (PSA) correlation along with biopsy of the enlarged and hypermetabolic axillary lymph nodes wasadvised. On questioning the patient after the scan, he was found be having mild difficulty in micturation and lumbago. The patient’s PSA level was found to be 4020 ng/mL and transrectal ultrasound (TRUS)-guided biopsy of the prostate revealed adenocarcinoma of the prostate with a Gleason score of 9 (5+4). The patient underwent subcapsular orchidectomy and was started on hormonal treatment with bicalutamide. With this treatment, the clinically palpable cervical, supraclavicular, and axillary lymphadenopathy was resolved within a month. A follow-up FDG PET/CT scan was performed 3 months after the baseline scan, which revealed complete resolution of the metabolically active lymphadenopathy (Figure 2) and the prostate lesion. The skeletal lesions showed reduced metabolic activity with no significant change in CT appearance. Overall, a good response to hormonal treatment was noticed on the scan. In view of the complete resolution of the FDG-avid lymphadenopathy after orchidectomy and hormonal treatment, we inferred the extensive nodal disease to be metastatic carcinoma of the prostate.

## 3. Discussion

Prostate carcinoma is known to spread via three mechanisms, namely, local extension, hematogenous dissemination, and lymphatic metastasis (2). Lymphatic spread to the obturator, hypogastric, iliac, presacral, and para-aortic nodes is a common route of metastasis. Cervical and supraclavicular lymph node involvement has been reported in 0.4% to 1% of all cases of metastatic prostate cancer (1). Generalized lymphadenopathy is a very unusual manifestation of metastatic prostate cancer (3). Rare cases describing enlargement of the cervical and supraclavicular lymph nodes as the initial presenting sign in patients with previously unrecognized prostatic carcinoma are available in the literature (4-6). The left-sided supraclavicular nodes were found to be considerably more commonly involved than those on the right side, presumably as a result of spread via the thoracic duct. A plausible explanation for this phenomenon is that tumor cells become lodged in these nodes by retrograde spread, as the supraclavicular nodes are located close to the entry of the thoracic duct into the left subclavian vein (4, 5). This presumption holds well in our case, as only the left-sided supra-diaphragmatic lymph node groups were involved.

FDG PET and FDG PET/CT do not play a major role in the diagnosis of prostate carcinoma ecause FDG uptake in a carcinoma may have a significant overlap with the uptake level in the normal gland or in benign prostate conditions (7, 8). Preliminary clinical studies have demonstrated that FDG PET may be useful in the evaluation of advanced androgen-independent disease in patients with high Gleason scores and serum PSA levels, in the detection of active osseous and soft tissue metastases, and in the assessment of response after androgen ablation and treatment with novel chemotherapies (9). FDG PET and PET/CT may also play a vital role inevaluation of the response to therapy (10). In particular, pilot human clinical studies have demonstrated that FDG accumulation in both the primary tumor and the metastatic sites decreases as early as 1 to 5 months after androgen ablation(9). Our case describes a similar role played by FDG PET/CT in monitoring the response to androgen ablation and hormonal treatment. Diagnosis is crucial even in advanced metastatic disease because of the responsiveness of this carcinoma to androgen ablation and hormonal therapy (10, 11).

In conclusion, we present a case of adenocarcinoma of the prostate initially presenting as generalized lymphadenopathy and mimicking lymphoma on FDG PET/CT. Our case suggests that in elderly men presenting with generalized lymphadenopathy, the diagnosis of metastatic prostatic carcinoma should not be overlooked even in the absence of typical urinary symptoms. Serum PSA correlation and digital rectal examination can be useful in early diagnosis in such cases. The establishment of a diagnosis of metastatic prostate carcinoma is important because even widespread prostate cancer may be responsive to hormonal treatment, as demonstrated in our case. In patients with a positive FDG PET/CT scan at the time of diagnosis, follow-up FDG PET/CT can play an important role in monitoring the response to treatment.
